# Soluble recombinant enterovirus 71 VP1 fused to truncated newcastle disease virus nucleoprotein elicits immune responses in mice

**DOI:** 10.1007/s42770-025-01784-w

**Published:** 2025-09-23

**Authors:** Suhaili Mustafa, Noraini Abd-Aziz, Khatijah Yusoff, Norazizah Shafee

**Affiliations:** 1 Department of Animal Science and Fisheries, Faculty of Agricultural and Forestry Sciences, Universiti Putra Malaysia Sarawak, Bintulu, Sarawak Malaysia; 2 Malaysia Genome and Vaccine Institute, National Institutes of Biotechnology Malaysia, Jalan Bangi, Kajang, Selangor Malaysia; 3 Department of Microbiology, Faculty of Biotechnology and Biomolecular Sciences, Universiti Putra Malaysia, Serdang, Selangor Malaysia

**Keywords:** Soluble protein, Enterovirus 71, Recombinant vaccine, Hand foot and mouth disease, Nucleocapsid protein

## Abstract

**Introduction:**

Enterovirus 71 (EV71) is a major causative agent of hand, foot, and mouth disease with potential neurological complications, highlighting the urgent need for effective vaccines. The viral protein 1 (VP1) contains major antigenic and neutralizing epitopes, making it a promising target for subunit vaccine development.

**Aims:**

This study evaluated the immunogenicity of a recombinant VP1 fragment (amino acids 198-297) fused to a truncated Newcastle Disease Virus nucleoprotein (NPt-VP1t).

**Materials and methodology:**

Soluble NPt-VP1t (SP) and insoluble NPt-VP1t (IB) versions of the protein were expressed in *E. coli*, purified, and verified by SDS-PAGE and Western blot using anti-VP1 and anti-NDV antibodies, confirming the ~59 kDa fusion protein. Adult female BALB/c mice were immunized intraperitoneally with SP, IB, or PBS control, with two booster doses at two-week intervals.

**Results:**

Results demonstrated that mice immunized with the SP formulation produced significantly higher anti-VP1 IgG reactivity than those receiving the IB or controls (*p* < 0.05). After the first booster (week 4), the antibody level in the SP was approximately 2-fold higher than the IB, with the highest OD readings observed at week 8 post-immunization. The SP maintained high antibody levels for at least two weeks post-booster. Splenocyte proliferation assays revealed that the SB group had a stimulation index (S.I.) of 0.826 ± 0.104, about 1.6 times greater than the IB group (*p* < 0.05). Cytokine profiling showed significantly elevated Th1 (IFN-γ, IL-2) and Th2 (IL-4, IL-6, IL-10) cytokines in both SP and IB groups compared to controls (*p* < 0.05), with IFN-γ levels markedly higher in vaccinated mice, indicating activation of both humoral and cell-mediated immunity. SDS-PAGE of the IB protein revealed contaminant bands, suggesting the actual amount of NPt-VP1t administered was lower than in the soluble formulation, potentially affecting its immunogenicity. All groups received formulations with Freund’s adjuvant, which may limit assessment of vaccine-specific safety.

**Conclusion:**

Overall, the SP recombinant NPt-VP1t protein elicited robust humoral and cellular immune responses in mice, outperforming the insoluble form. These findings support the immunogenic potential of SP as a subunit vaccine candidate for EV71. Future studies should include viral challenge and neutralization assays to confirm protective efficacy.

**Supplementary Information:**

The online version contains supplementary material available at 10.1007/s42770-025-01784-w.

## Introduction

Enterovirus A71 (EV-A71), a member of the *Picornaviridae* family, is a primary causative agent of hand, foot, and mouth disease (HFMD), predominantly affecting infants and young children [1]. Although infections in adults are often mild or asymptomatic, EV-A71 can cause severe neurological complications in children under five, including meningitis, encephalitis, and acute flaccid paralysis [2]. Due to its significant morbidity and mortality, particularly in the Asia-Pacific region, EV-A71 has become a critical focus of public health research [1]. Since its discovery in 1969, multiple outbreaks have been reported globally, with Southeast Asia being especially impacted. A major outbreak in Cambodia in 2012 resulted in nearly 60 child fatalities, while between 2008 and 2013, China recorded over nine million HFMD cases, with EV-A71 accounting for approximately 90% of the fatal outcomes [3]. Malaysia experienced a significant outbreak in 1997, causing 29 pediatric deaths in Sarawak [4]. In 2016, Malaysian surveillance data indicated a sharp weekly rise in HFMD cases, with Selangor reporting the highest incidence nationwide. Despite the serious public health threat, no universally effective antiviral therapy or vaccine is currently available for EV-A71. Preventive measures rely mainly on hygiene practices and isolation of infected individuals. Vaccine development has thus become a priority, with multiple platforms under investigation, including inactivated virus, live attenuated virus, virus-like particles (VLPs), subunit proteins, and DNA-based vaccines [1]. In 2015 and 2016, two inactivated EV-A71 vaccines were licensed in China, demonstrating high efficacy in clinical trials [5, 6]. However, potential risks related to incomplete inactivation or reversion to virulence render subunit vaccines a safer alternative.

Among EV-A71’s structural proteins, viral protein 1 (VP1) is a key neutralizing antigen known for its strong immunogenicity and presence of major antigenic sites, making it an ideal subunit vaccine candidate [7]. The C-terminal region of EV-A71 VP1 (amino acids 198–297), which contains the conserved SP70 neutralizing epitope and other antigenic determinants, was selected for this construct because it induces potent virus-neutralizing antibodies in both animal models and human convalescent sera [7, 8]. This fragment is structurally stable and less prone to conformational changes than the full-length VP1 protein, making it more suitable for recombinant expression. To further enhance its immunogenicity, the truncated nucleocapsid protein (NPt) from Newcastle Disease Virus was employed as a carrier. Previous studies have demonstrated that NPt is intrinsically immunogenic, self-assembles into multimeric structures, and significantly enhances both humoral and cellular immune responses when fused to weak antigens [8–11]. Recombinant constructs incorporating the N- or C-terminal domains of VP1 fused to NPt, with one promising construct, NPt-VP1_198 − 297_, showing good immunogenic potential in mice, although predominantly expressed in insoluble form [12]. However, antigen solubility can significantly influence protein folding, epitope exposure, and overall immune recognition. Thus, this study aims to evaluate the immunogenic response elicited by the soluble form of NPt-VP1t in a murine model.

## Materials & methods

### Source of recombinant protein and antigen

*E. coli* Rosetta-gami cells carrying the expression vector pTrcHis2, encoding a truncated nucleocapsid protein gene (NPt; 1173 bp, representing 80% of the N-terminal nucleotides of the NPfl gene) from Newcastle disease virus (NDV) strain AF2240 (*GenBank* accession no. AF284646) fused to a truncated viral protein 1 segment (VP1t; 300 bp, corresponding to amino acids 198–297 of VP1) from human enterovirus 71 (EV71) strain MY104/9/SAR/97 (*GenBank* accession no. AF376072), were obtained from the Virology Laboratory, Department of Microbiology, Faculty of Biotechnology and Biomolecular Sciences, Universiti Putra Malaysia. This recombinant construction, designated pTrcHis2-NPt-VP1_198-297_, plasmid construction was performed as previously described by Mustafa et al., 2020 [12]. Detailed verification data, including plasmid schematic, restriction digestion, PCR amplification, and sequence alignment, are provided in Supplementary Figures S1–S5.

### Recombinant protein expression and detection in E. coli using IPTG induction

Bacterial clones harboring the NPt-VP1t constructs were cultured overnight in 5 mL LB medium with appropriate antibiotics (100 µg/mL ampicillin or kanamycin) at 37 °C, 250 rpm. A 1 mL aliquot was then transferred into 40 mL fresh Terrific Broth (TB) containing the same antibiotics, mixed thoroughly, cultures were grown at 37 °C, 250 rpm until OD_6_₀₀ reached 0.6–0.8, then induced with 1.0 mM IPTG and incubated at 37 °C for 7 h. Cells were harvested by centrifugation (6,000 × g, 4 °C), resuspended in 100 µL PBS (20 mM sodium phosphate, 0.9% NaCl, pH 7.4), and mixed with 0.03 volumes of 6X sample buffer (2.8X Tris-Cl/SDS (pH 6.8), 10% (w/v) SDS, 30% (v/v) glycerol, 0.12% (w/v) bromophenol blue, 6% (v/v) 2-mercaptoethanol). The final mixture corresponded to a 1× working concentration. Samples were boiled for 15 min, and 15 µL was analyzed by 12% SDS-PAGE, followed by PVDF membrane blotting with anti-VP1 (Abcam, UK) or anti-His (Sigma-Aldrich, USA) antibodies (Table 1). Protein band intensities were quantified using a calibrated densitometer.

**Table 1:** Dilution of antibodies used for Immunoblotting

Antibodies	Dilution in 1X TBS
Anti-His	1:5000
Anti-VP1 Anti-NDV	1:2000 1:2000

### Purification of recombinant NPt-VP1t protein

#### Purification of soluble fraction (SP)

Recombinant *E. coli* cultures (2 L) were grown at 37 °C and induced with 1.5 mM IPTG. Following cell harvest and lysis, the clarified lysate was subjected to immobilized metal affinity chromatography (IMAC) using a HisTrap HP column (GE Healthcare, Sweden). The column was pre-equilibrated with binding buffer (20 mM sodium phosphate, 500 mM NaCl, 30 mM imidazole, pH 7.4). After sample application, non-specifically bound proteins were removed by washing with 12 column volumes of the same buffer. Elution was optimized using a stepwise imidazole gradient (60–500 mM), and final elution was performed with 500 mM imidazole. The eluted protein was dialyzed against 20 mM sodium phosphate buffer containing 200 mM NaCl (pH 8.0) for 48 h at 4 °C using SnakeSkin^®^ dialysis tubing (10 kDa MWCO; Thermo Scientific, USA) and concentrated with Vivaspin centrifugal filters (100 kDa MWCO; Sartorius Stedim, Germany).

#### Purification of inclusion body fraction (IB)

Purification of the insoluble fraction followed a modified method [13]. Cultures were induced with 1.0 mM IPTG, harvested, and lysed. Pellets were resuspended in 0.03 volume of lysis buffer with 1% Triton X-100, incubated on ice (10 min), and centrifuged at 12,000 × g for 20 min at 4 °C. The wash was repeated without detergent. Pellets were solubilized in denaturing buffer (20 mM sodium phosphate, 500 mM NaCl, 8 M urea, 0.3 mM GSH, 5% glycerol, pH 7.4) and incubated at room temperature for 2 h. After centrifugation, the supernatant was loaded onto a HisTrap HP column equilibrated with denaturing binding buffer. The column was sequentially washed with buffers containing 8 M to 2 M urea and supplemented with 3 mM GSH, 0.3 mM GSSG, and 30 mM imidazole to remove contaminants. Elution was optimized with a stepwise imidazole gradient (40–500 mM) in 2 M urea-containing buffer. Final elution was performed with buffer containing 2 M urea, 5% glycerol, 3 mM GSH, and 0.3 mM GSSG. Eluted proteins were dialyzed overnight at 4 °C against 1 M urea in 20 mM sodium phosphate buffer (200 mM NaCl, 5% glycerol, pH 8.0) and concentrated using Vivaspin columns (Sartorius Stedim, Germany). No direct assessment of refolding efficiency or conformational integrity was performed.

### Protein quantification

Protein concentrations were determined by the Bradford assay [14] using bovine serum albumin (BSA; USB, Canada) as the standard. Absorbance was measured at 595 nm using an iMark Microplate Reader (Bio-Rad, USA). For analysis, 5 µL of sample was mixed with 15 µL of TBS (0.1 M Tris, 0.15 M NaCl, pH 8.0) and 200 µL of Bradford reagent. All measurements were performed in duplicate, and concentrations were calculated based on a standard curve generated from 0 to 10 µg BSA.

### Immunogenicity studies

All animal procedures were conducted with approval from the Animal Care and Use Committee, Faculty of Veterinary Medicine, Universiti Putra Malaysia (AUP No. 10R84), and followed guidelines from the Malaysian Association for Accreditation of Laboratory Animal Care and the Code of Practice for the Care and Use of Animals in Research. The immunogenicity study involved three groups of mice. The control group (*n* = 6) received PBS emulsified with 50% complete Freund’s adjuvant (Sigma, USA) for primary injection and 50% incomplete Freund’s adjuvant (Sigma, USA) for two booster injections. The SP group (*n* = 8) was immunized with 10 µg of purified soluble NPt-VP1t protein in PBS, emulsified with the same adjuvant schedule. The IB group (*n* = 8) received 10 µg of total protein from the first elution fraction (E1) of the denaturing purification after dialysis, which contained the NPt-VP1t protein along with co-eluted proteins. It is also formulated with complete Freund’s adjuvant for the primary injection and incomplete Freund’s adjuvant for the two booster injections. Densitometry analysis of SDS-PAGE gels indicated that the ~ 59 kDa NPt-VP1t band represented approximately 70% of the total protein in E1, corresponding to an estimated 7 µg of IB per dose, with the remaining ~ 3 µg comprising other proteins. Blood samples were collected via tail vein bleeding at weeks 0, 2, 4, 6, 8, 9, and 10. Samples were kept overnight at 4 °C to allow clotting, then centrifuged (3,000 rpm, 20 min) to separate sera, which were stored at − 20 °C until analysis. The immunization protocol followed [8]. Body weights of immunized mice were monitored biweekly and recorded weekly to assess potential physiological effects of immunization. Data were presented graphically.

### Determination of anti-VP1 and anti-NP antibody reactivity by indirect ELISA

Anti-VP1 antibody reactivity in mouse sera were determined using an indirect ELISA with purified VP1 protein (1.5 µg/mL in 1X TBS, pH 8.0) as the coating antigen. Each well of a high-binding 96-well ELISA plate was coated with 100 µL of the antigen and incubated overnight at 4 °C in a sealed, humidified chamber with shaking.

The next day, unbound antigen was removed, and wells were washed three times with TBS-T (0.05% Tween-20), followed by blocking with 250 µL of diluted milk (1:20 in TBS) for 2 h at 4 °C. After washing, 100 µL of mouse sera (1:50 dilution in TBS) were added in duplicates and incubated for 1 h at room temperature with shaking. Plates were washed again and incubated with 100 µL of HRP-conjugated anti-mouse IgG (1:1000 dilution in TBS) for 1 h. Following a final wash, 100 µL of OPD substrate (0.4 mg/mL in 0.05 M citrate phosphate buffer, pH 5.0, with 20 µL of 30% H_2_O_2_) was added to each well. After 5 min of incubation at room temperature, the reaction was stopped with 100 µL of 3 M sulfuric acid. Absorbance was measured at 490 nm using an iMark Microplate Reader (Bio-Rad, USA).

### Cellular immune response and T-cell proliferation assay

At week 9 post-immunization, five mice from each group (soluble protein-immunized, insoluble protein-immunized, and control) were sacrificed to assess cellular immune responses. Spleens were aseptically harvested, and single-cell suspensions of splenocytes were prepared by passing the tissue through 70 μm cell strainers using a sterile 10 mL syringe plunger. The splenocyte suspensions were centrifuged at 1,000 rpm for 10 min at 4 °C. Red blood cells were lysed twice using 5 mL of lysis buffer (0.84% NH_4_Cl, 0.1% NaHCO_3_, 1.8% EDTA), followed by incubation for 5 min at 4 °C and centrifugation. The splenocytes were subsequently washed with 1X PBS-EDTA and centrifuged at 250 × g for 10 min at 4 °C. Finally, the cells were resuspended in 5 mL of complete DMEM supplemented with 10% foetal bovine serum (FBS). T-cell proliferation was evaluated using a BrdU Cell Proliferation ELISA Kit (Calbiochem, Germany). Splenocytes (2 × 10⁵ cells/well) were seeded into sterile 96-well tissue culture plates containing DMEM with 10% FBS and 1% antibiotic-antimycotic and stimulated with 3 µg of purified VP1 protein or Concanavalin A (Con A) as a positive control. Unstimulated wells served as background controls. Cultures were incubated at 37 °C with 5% CO_2_ for 72 h. Following incubation, 20 µL of BrdU labelling solution (1:2000 dilution) was added to each well, and the cells were incubated for an additional 18 h. After harvesting by centrifugation (250 × g, 10 min), the supernatants were removed and 200 µL of Fixative/Denaturing Solution was added to each well for 30 min at room temperature. After discarding the fixative, 100 µL of anti-BrdU antibody (1:100 dilution) was added and incubated for 1 h. Plates were washed three times with 1X Wash Buffer before adding 100 µL of HRP-conjugated goat anti-mouse IgG (1:2000 dilution), followed by 30 min of incubation at room temperature. After final washing steps, colour development was initiated by incubation in the dark for 15 min, followed by the addition of 100 µL of Stop Solution. Absorbance was measured at 490 nm using an ELISA microplate reader (Bio-Rad Model 550). T-cell proliferation was expressed as the stimulation index (S.I.), calculated by dividing the mean absorbance of stimulated splenocytes by the mean absorbance of the background controls.

### Cytokine assay

Mouse Th1/Th2 cytokine standards were prepared by reconstituting lyophilized standards with 2 mL of Assay Diluent to create the top standard (5,000 pg./mL), which was allowed to equilibrate for 15 min at room temperature. A series of eight 1:2 serial dilutions were then prepared to obtain final concentrations of 2,500, 1,250, 625, 312.5, 156, 80, 40, and 20 pg./mL. An additional tube with only Assay Diluent served as the 0 pg./mL negative control. Capture beads for cytokine detection were prepared by vertexing each bead suspension and mixing 10 µL of each bead type per assay tube into a single tube, referred to as “Mixed Capture Beads.” For example, for 18 assay tubes, 180 µL of each cytokine-specific capture bead was used. Splenocytes (2 × 10⁵ cells/well) from mice immunized with soluble or insoluble NPt-VP1t, as well as control groups, were cultured in 96-well plates in DMEM supplemented with 10% FBS and 1% antibiotic-antimycotic. Cells were stimulated with 3 µg of purified VP1 protein and incubated at 37 °C with 5% CO_2_ for 72 h. After incubation, culture supernatants were collected for cytokine analysis using the BD Biosciences Cytometric Bead Array (CBA) Mouse Th1/Th2 Cytokine Kit, which detects IL-2, IL-4, IL-6, IL-10, IFN-γ, and TNF. Fifty microliters each of mixed capture beads, standard dilutions or samples, and PE-conjugated detection reagent were added to the assay tubes and incubated for 2 h at room temperature in the dark. After incubation, 1 mL of wash buffer was added, and tubes were centrifuged at 200 × g for 5 min. The supernatant was discarded, and the beads were washed again before resuspending the pellet in 300 µL of wash buffer. Samples were analysed using a BD FACS Canto II Flow Cytometer, and cytokine concentrations were determined using FCAP Array™ software. Calibration curves generated from standard dilutions were used to quantify cytokine levels. The theoretical detection limits for the assay were: IL-2 (0.1 pg./mL), IL-4 (0.03 pg./mL), IL-6 (1.4 pg./mL), IFN-γ (0.5 pg./mL), TNF (0.8 pg./mL), and IL-10 (16.8 pg./mL).

### Statistical analysis

Statistical analysis was performed using Graph Pad Prism 6.0 and Windows Microsoft Excel 2010. Statistical analysis was performed using analysis of variance (ANOVA). *P* value less than 0.05 was considered significant. GS-800 Calibrated Densitometer (Bio-Rad, USA), Quantity One and PD Quest analysis software was used to determine the density of the bands on x-ray films. Student t-test was used to analyze the experimental data in this study. All experiment was performed using appropriate technical and biological replicates at least three replicates. Error bar represents the ± standard error of the mean (SEM).

## Results

The pTrcHis2-NPt-VP1_198 − 297_ expression construct used in this study has been described and validated in our earlier work [11]. Briefly, it consists of the truncated nucleocapsid protein (NPt; 1173 bp) from NDV strain AF2240 (*GenBank* accession no. AF284646) fused to the C-terminal 100 amino acids (300 bp) of EV71 VP1 (VP1t; amino acids 198–297) from strain MY104/9/SAR/97 (*GenBank* accession no. AF376072). Verification data, including schematic diagram, restriction digestion patterns, PCR amplification results, and BLAST sequence alignment, are presented in Supplementary Figures S1–S5.

### Purification of the soluble fraction of the NPt-VP1t recombinant protein

**Fig. 1:**
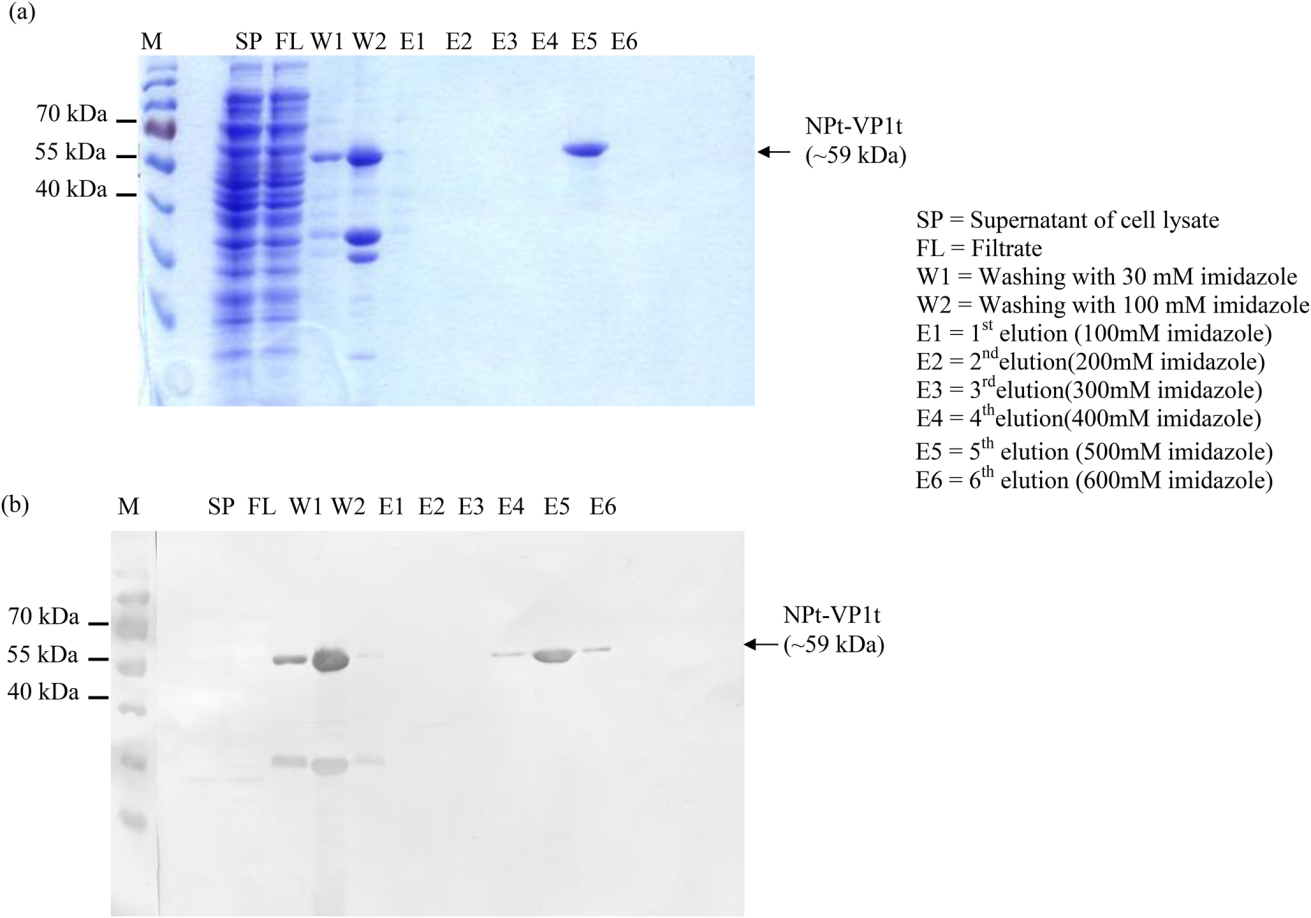
Purification of SP fraction of the NPt- VP1_198-297_ proteins. The purification was done using a HisTrap HP column as described in the Materials and Methods. (**a**) Aliquots of each purification step were electrophoresed on a 12% SDS-PAGE and stained. (**b**) A replicate gel was transferred onto membrane and probed with an anti-His antibody. M: pre-stained protein molecular weight markers

The purification of the SP using a HisTrap HP column was confirmed by SDS-PAGE and immunoblotting (Fig. 1). As shown in Fig. 1, a prominent band corresponding to the expected molecular weight of approximately 59 kDa [11] was detected in the fifth elution fraction (E5), indicating successful elution of the target recombinant protein. Minimal protein was observed in the wash fractions (W1 and W2), suggesting effective removal of non-specifically bound proteins. Immunoblotting using an anti-His antibody further verified the identity of the purified protein. A distinct band at ~ 59 kDa was detected in the same elution fraction (E5), corresponding to the SDS-PAGE result, confirming the presence of the His-tagged NPt-VP1t protein. Additional faint bands were observed in other elution lanes (e.g., E1 and E3), but the strongest signal was seen in E5, where 500 mM imidazole was used, supporting the effective elution of the protein at this concentration.

### Urea solubilization, purification and refolding of NPt-VP1t

**Fig. 2:**
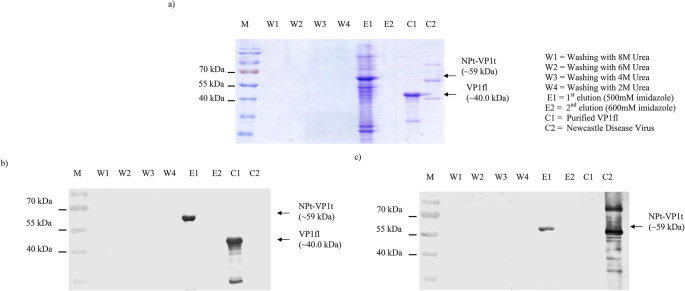
Purification and detection of the IB of the NPt-VP1t protein. The purification was carried out under denaturing conditions using 8 M urea and a HisTrap HP column, as described in the Materials and Methods. (**a**) Aliquots of each purification step were electrophoresed on a 12% SDS-PAGE and stained with Coomassie Brilliant Blue R-250. (**b**) A replicate gel was transferred onto PVDF membrane and probed with (**b**) rabbit anti-VP1 antibody and (**b**) rabbit anti-NDV antibody

The IB was purified under denaturing conditions using 8 M urea and immobilized metal affinity chromatography (IMAC) with a HisTrap HP column. SDS-PAGE (Fig. 2a) showed a clear ~ 59 kDa band in the first elution (E1, 500 mM imidazole), indicating recovery of the target protein. Minimal protein was detected in wash fractions (W1–W4, 8–2 M urea), confirming efficient removal of contaminants. A weaker ~ 59 kDa band appeared in the second elution (E2, 600 mM imidazole). Lane C1 showed a ~ 40 kDa band corresponding to full-length VP1 (VP1fl), while lane C2 (NDV control) lacked the 59 kDa band, confirming purification specificity. All steps (20 µl each) were analyzed by SDS-PAGE, and no protein was detected in W1–W4, suggesting strong His-tag binding. Two replicate sets were also analyzed by Western blot using anti-VP1 and anti-NDV antibodies. The blot probed with anti-VP1 antibodies showed a distinct band in the E1 lane (Fig. 2b), confirming the presence of the NPt-VP1t construct, supported by detection of VP1fl in the C1 positive control. Similarly, the blot probed with anti-NDV antibodies showed a single band in E1 (Fig. 2c), indicating that the construct includes the NPt carrier, an NDV-derived protein. The C2 positive control confirmed specificity with bands corresponding to purified NDV proteins. These results confirmed recovery of the ~ 59 kDa NPt-VP1t protein, although co-purified contaminants were also present in the elution fraction, consistent with the expected ~ 59 kDa size reported previously [8, 9, 15]. The IB fraction was subsequently subjected to stepwise dialysis to promote refolding. However, no confirmatory analyses (e.g., circular dichroism spectroscopy) were performed to assess refolding efficiency or conformational integrity, and the preservation of native epitopes could not be verified. The dialyzed E1 fraction contained visible contaminant proteins (Fig. 2a), which may have contributed to the immune responses observed. Based on gel band intensity, the actual amount of NPt-VP1t in the injected 10 µg E1 fraction was estimated to be approximately 7 µg, with the remainder consisting of other proteins.

### Immunogenicity of NPt-VP1t in mice

**Fig. 3:**
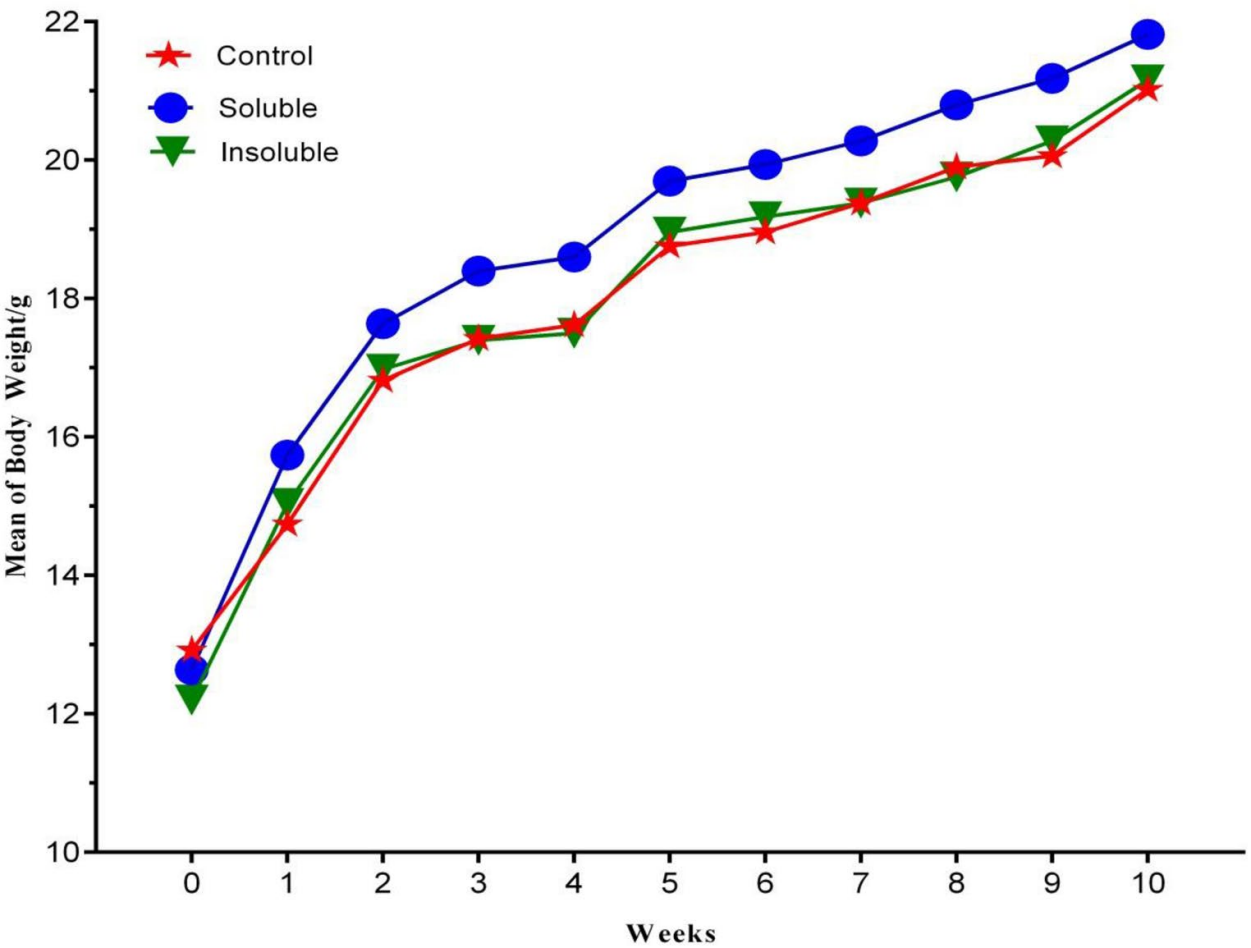
Mice body weight after immunization with recombinant vaccine. Weight of the mice was for weight every two weeks and recorded

To evaluate the immunogenicity of the purified recombinant NPt-VP1t protein, mice immunogenicity studies were performed. Mice weight was monitored throughout the immunization period. Mice sera were collected at selected intervals and the presence of antibody towards NPt-VP1t was tested. To evaluate the specific types of immune response produced by mice, splenocyte proliferation and cytokine production were also evaluated. Body weights were measured on the day of dosing (Day 0) immediately before immunization and then measured again after every immunization. Results show that there were no significant changes in body weight for the immunized mice when compared to the control groups (Fig. 3). The lack of difference suggested that of the mice with the recombinant proteins did not significantly interfere with their normal bodily processes.

**Fig. 4:**
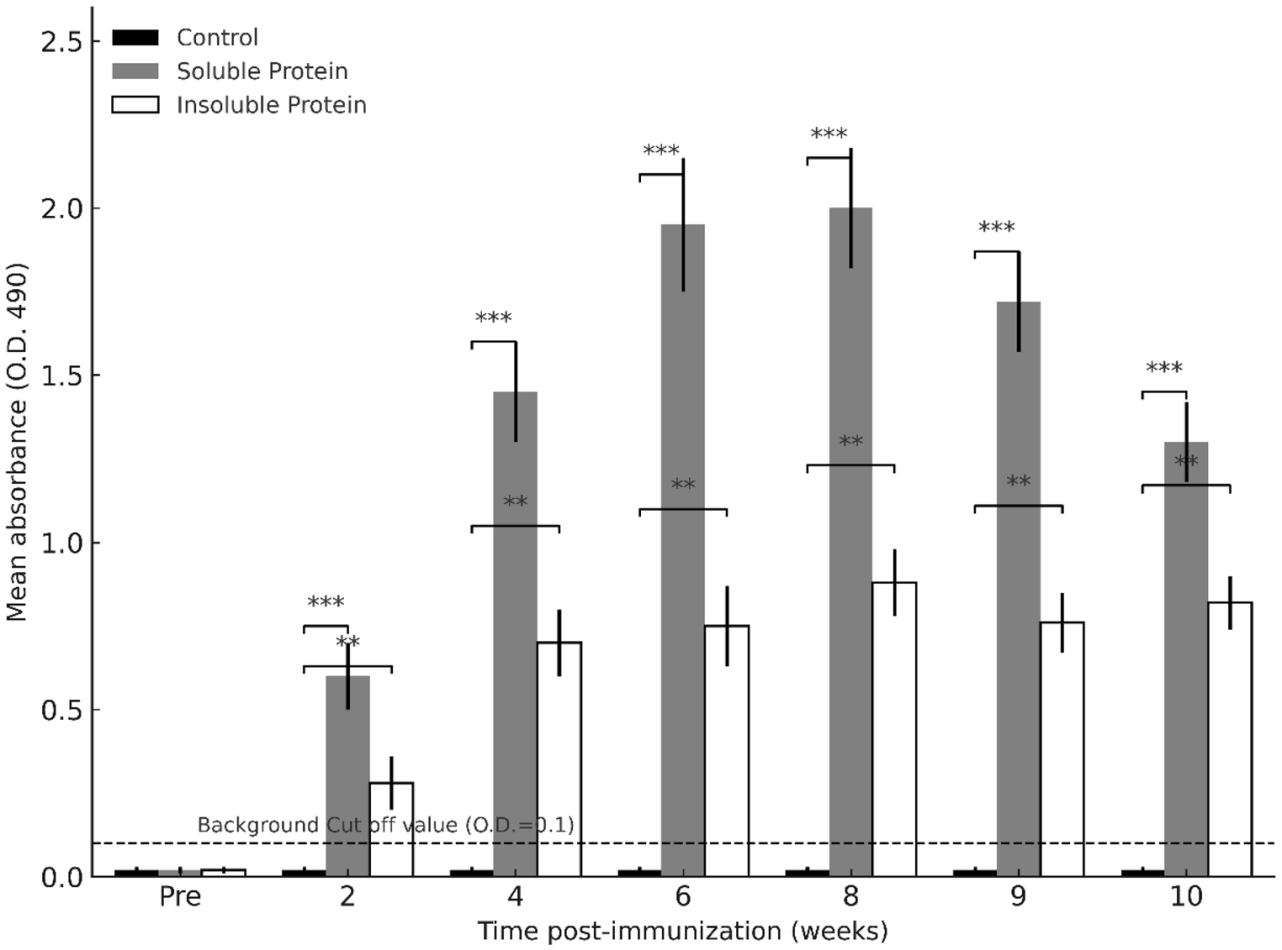
Total anti-VP1 IgG antibodies in mice sera following immunization with soluble and insoluble recombinant NPt-VP1t proteins. Mice (*n* = 5 per group) were immunized at weeks 0, 4, and 8 (arrows). Sera were collected at the indicated time points and antibody levels were determined by indirect ELISA. Bars represent mean ± SD. The dashed horizontal line represents the background cut-off value (O.D. = 0.1). Control = PBS; Soluble Protein ; Insoluble Protein. Statistical differences compared with the control group are indicated (*p* < 0.01 = **, *p* < 0.001 = ***; one-way ANOVA with Tukey’s post hoc test)

Results showed that the NPt-VP1t protein elicited anti-VP1 IgG responses, with distinct differences between the soluble and insoluble formulations (Fig. 4). Individual data points are presented alongside mean ± SD to illustrate variability within groups, and statistical differences are indicated. Pre-immune background mice sera were used as a positive cut off value, and negative control readings remained close to background throughout the study. The anti-VP1 IgG reactivity for SP group showed a significant (*p* < 0.05) increase in anti-VP1 IgG reactivity after the primary immunization, which was further enhanced following each booster when compared to the IB group. After the first booster in week 4 and immediately before the second booster injection, anti-VP1 IgG reactivity for SP was 2 times higher than the antibody reactivity of IB. Peak responses were observed in week 8 for both groups, but the SP consistently maintained significantly higher antibody reactivity (*p* < 0.05). Reactivity remained elevated for at least two weeks after the final booster before gradually declining; however, SP responses remained above those of the IB group until the end of the study. Interestingly, while the SP formulation showed a slight decrease after week 9, the IB group exhibited a modest increase, though still at substantially lower levels than the SP group.

### Cellular-mediated immunity

**Fig. 5:**
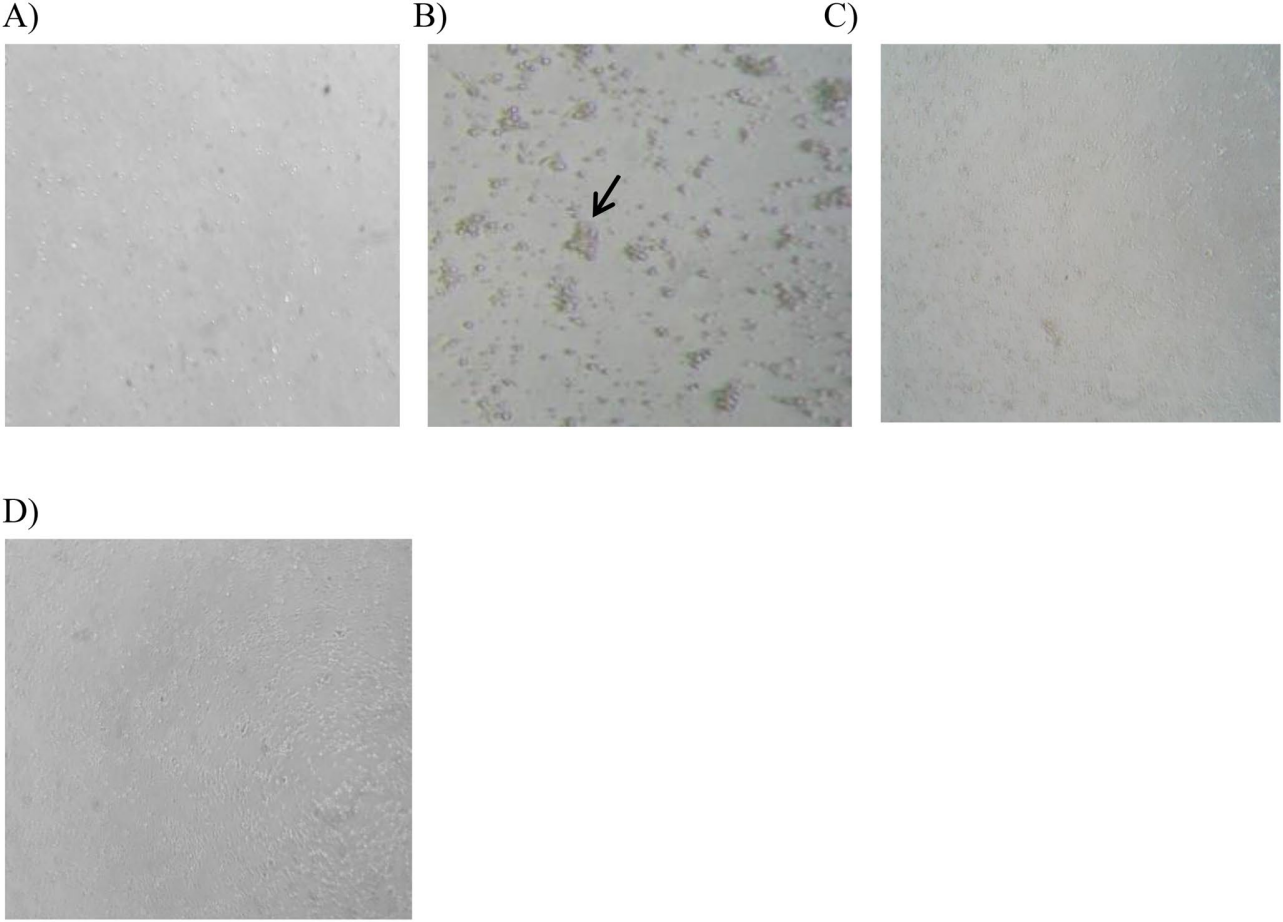
Splenocytes activation of control mice after treatment with ConA stimulator and VP1 antigen. (**A**) splenocytes before treatment; (**B**) ConA-treated splenocytes; (**C**) VP1-treated splenocytes. (**D**) Untreated splenocytes after 72 h incubation. Arrow indicates splenocyte clumping after treatment. Magnification = 100X

**Fig. 6:**
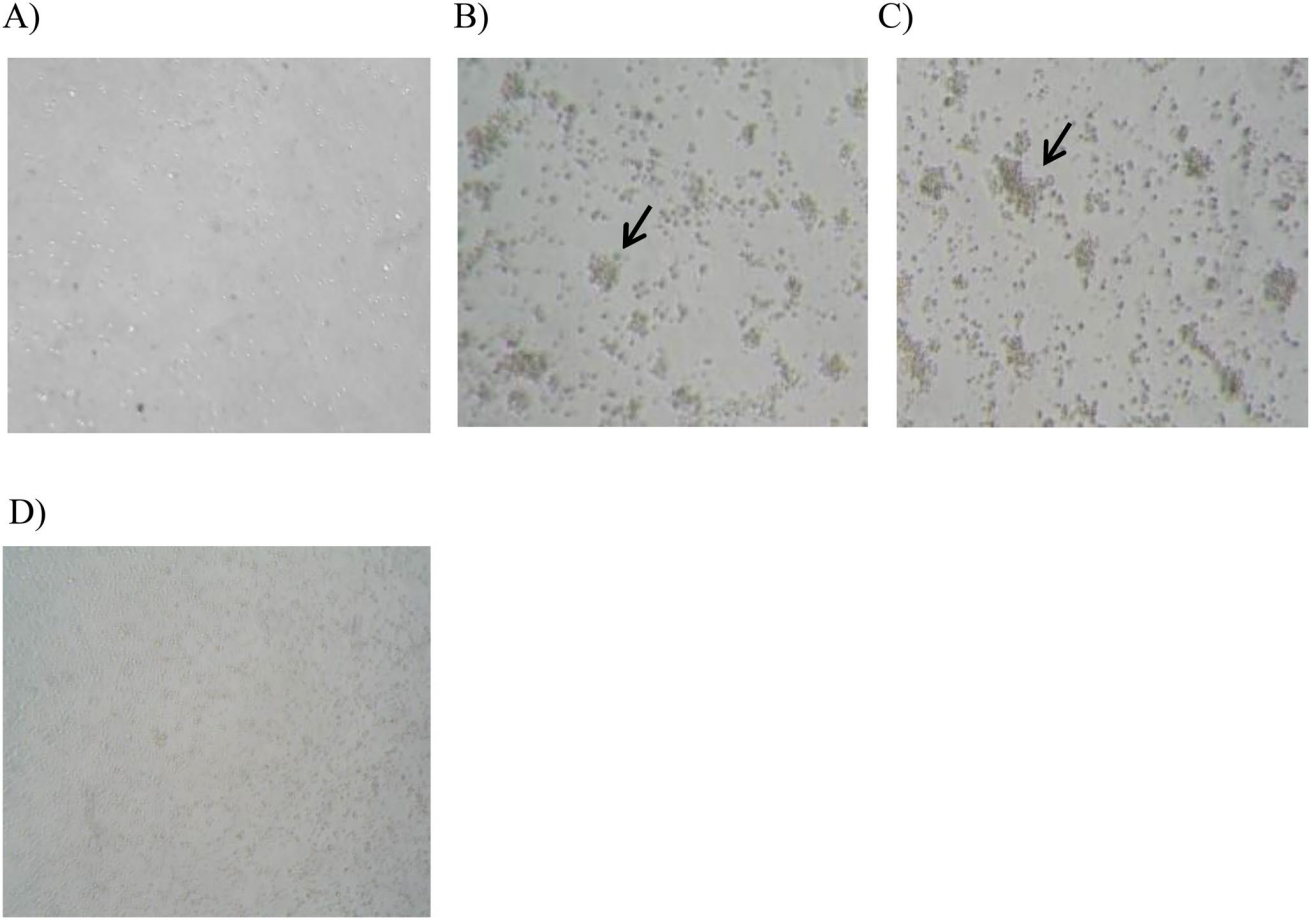
Splenocytes activation soluble protein immunized mice after treatment with ConA stimulator and VP1 antigen. (**A**) splenocytes before treatment; (**B**) ConA-treated splenocytes; (**C**) VP1-treated splenocytes. (**D**) Untreated splenocytes after 72 h incubation. Arrow indicates splenocyte clumping after treatment. Magnification = 100X

**Fig. 7:**
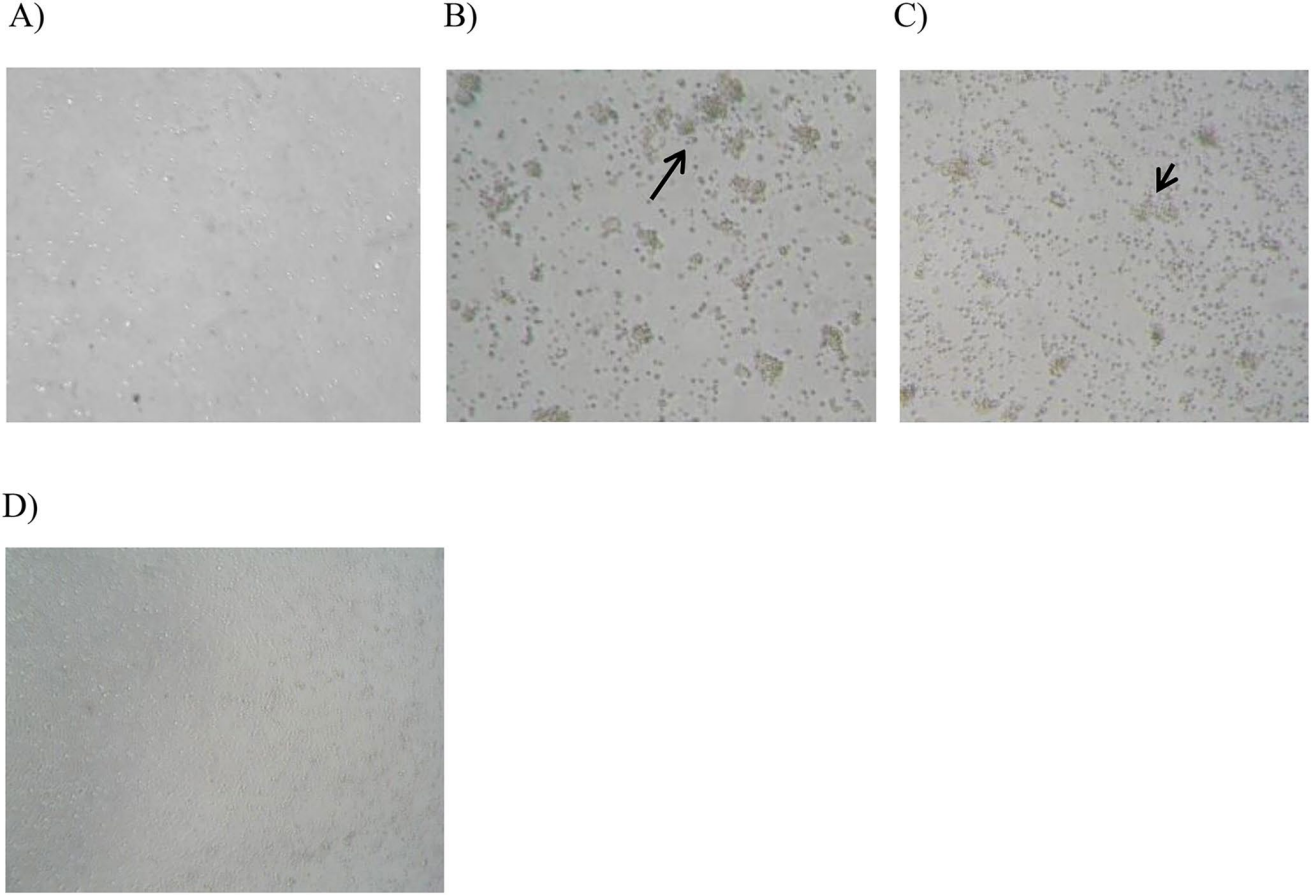
Splenocytes activation insoluble protein immunized mice after treatment with ConA stimulator and VP1 antigen. (**A**) splenocytes before treatment; (**B**) ConA-treated splenocytes; (**C**) VP1-treated splenocytes. (**D**) Untreated splenocytes after 72 h incubation. Arrow indicates splenocyte clumping after treatment. Magnification = 100X

**Fig. 8:**
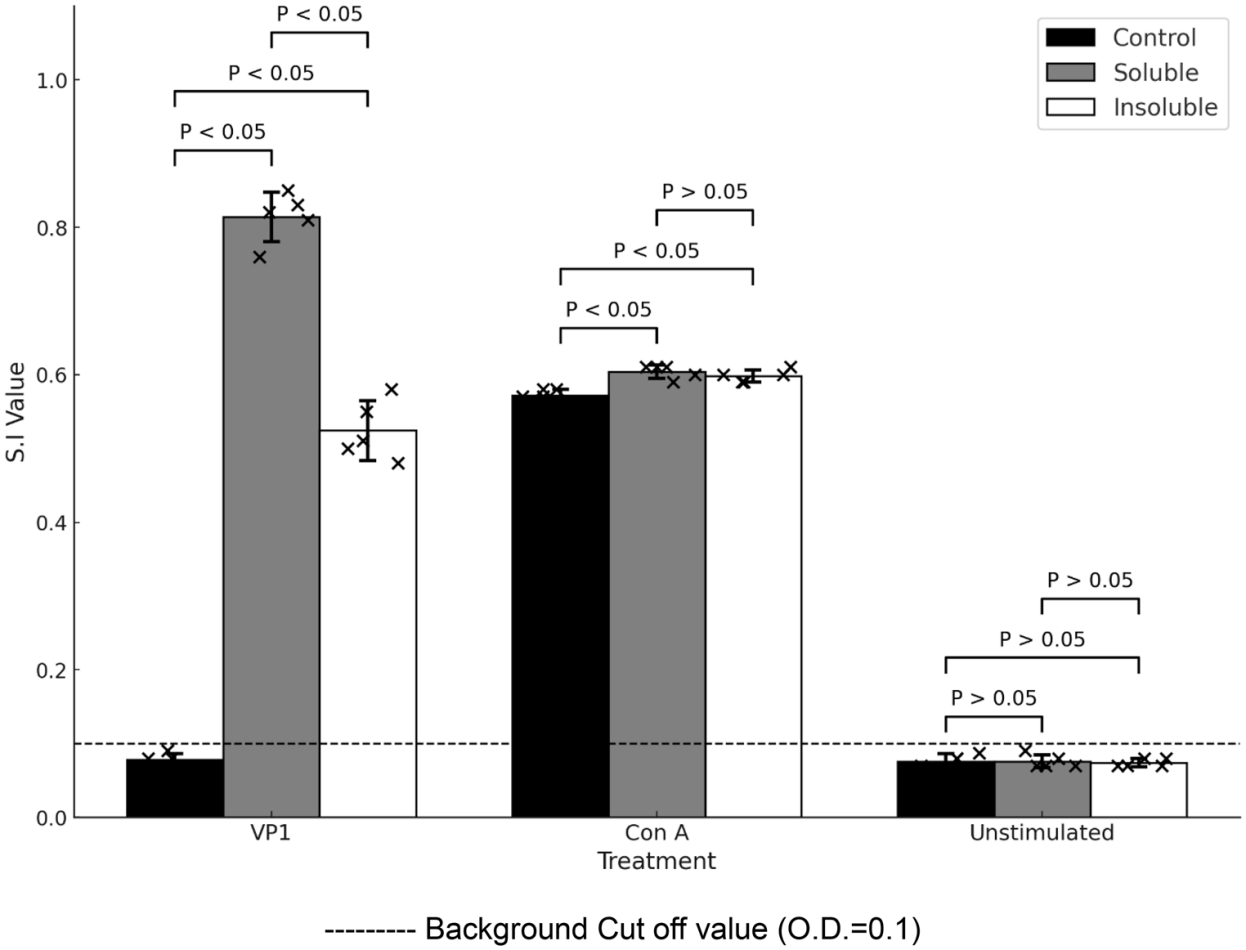
T-cell proliferation of splenocytes from soluble protein-immunized, insoluble protein-immunized,and control mice (*n* = 5) at week 9 post-immunization. Cells were stimulated with 3 µg/mL VP1 protein or Concanavalin A (ConA, positive control), with unstimulated wells as background controls. Proliferation was measured using a BrdU ELISA, and stimulation index (S.I.) was calculated as the ratio of stimulated to unstimulated OD490 values. Bars show mean ± SD; symbols indicate individual values. Statistical analysis was performed using one-way ANOVA followed by Tukey’s post hoc test; *p* < 0.05 was considered significant

The results indicate that splenocyte activation occurred in response to both VP1 antigen and ConA stimulation, as evidenced by cluster formation of suspension cells (Figs. 5B and 6B–C, and 7B–C, arrows). ConA stimulation did not result in significant differences (*p* > 0.05) in splenocyte proliferation among the control, soluble, and insoluble NPt-VP1t immunized groups (Fig. 8, ConA bars), confirming similar baseline responsiveness. However, upon VP1 stimulation, both soluble and insoluble NPt-VP1t immunized groups showed significantly enhanced T-cell proliferation compared to controls (*p* < 0.05), with the soluble protein group eliciting a stronger response. The stimulation index (S.I) for the soluble group (0.826 ± 0.104) was approximately 1.6 times higher than that of the insoluble group, indicating superior immunogenicity. These findings suggest that NPt-VP1t effectively induces a VP1-specific T-cell response, with the soluble form being more potent.

### Cytokines production by VP1 antigen-induced splenocytes

**Fig. 9:**
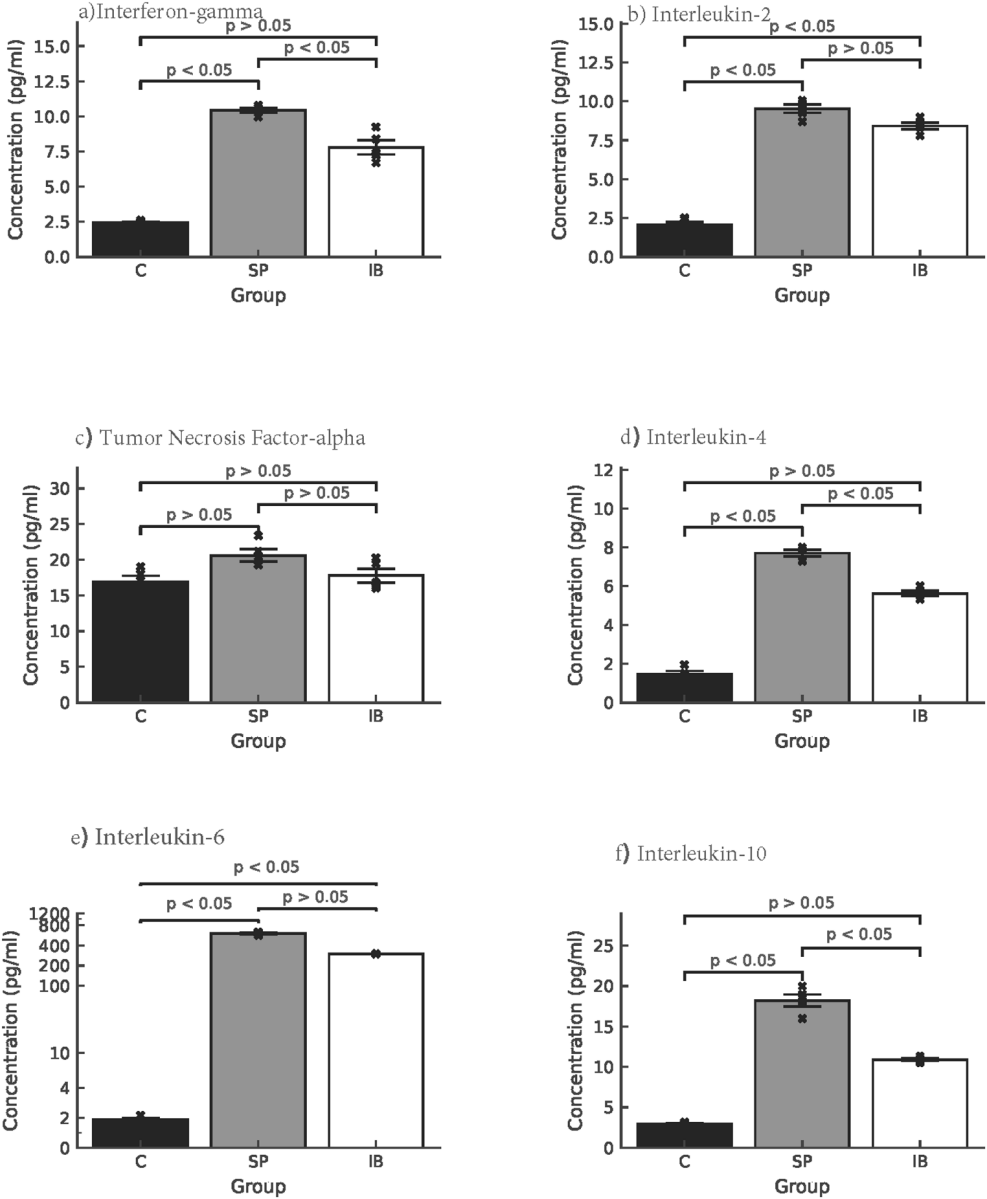
Cytokine levels in mice sera at week 10 post-immunization. Bars represent mean ± SD for control (C), soluble protein (SP), and insoluble protein (IB) groups. SP induced significantly higher IFN-γ, IL-2, IL-4, IL-6, and IL-10 than C and IB (*p* < 0.05), except TNF-α, which showed no significant differences. Significance was determined by one-way ANOVA with Tukey’s post hoc test

Cytokine profiling (Fig. 9) revealed that mice immunized with the SP group produced significantly higher levels of IFN-γ, IL-2, IL-4, IL-6, and IL-10 compared to the IB group and control group (*p* < 0.05 for all). For IFN-γ, IL-2, and IL-6, SP group levels were significantly greater than both IB and control groups, whereas IB group levels did not differ significantly from controls. IL-4 and IL-10 production in the SP group was significantly higher than in both IB and control groups, while IB group responses remained similar to controls. TNF-α levels did not differ significantly among groups, indicating that vaccination did not induce excessive systemic inflammation. These patterns are consistent with a balanced Th1/Th2 immune response induced by the SP group, with minimal inflammatory skewing.

## Discussion

Recombinant protein expression in *E. coli* frequently leads to the formation of inclusion bodies, particularly for fusion proteins and viral antigens. This phenomenon arises due to differences in the intracellular environment of *E. coli* compared to the protein’s native host, including pH, redox potential, and folding machinery, as well as factors such as overexpression, hydrophobic interactions, and structural complexity [16, 17]. While inclusion bodies can be advantageous for protein enrichment, ease of isolation, and protection from proteolytic degradation [18–20], proper solubilization and refolding are required to restore native conformation and immunogenicity. In this study, the NPt-VP1t fusion protein was predominantly expressed as IB necessitating purification under denaturing conditions followed by dialysis. The insoluble antigen used for immunization was the dialyzed first elution fraction (E1) from the HisTrap IMAC purification, as shown in Fig. 2a. This fraction contained the ~ 59 kDa NPt-VP1t band together with several co-purified *E. coli* proteins. No additional purification or confirmatory refolding analysis (e.g., circular dichroism) was performed, and the preservation of native epitopes could not be verified [21].

Immunization was conducted with 10 µg of total protein from the dialyzed E1 fraction, corresponding based on densitometric analysis to an estimated 7 µg of NPt-VP1t and ~ 3 µg of other proteins. Thus, the actual NPt-VP1t dose in the IB group was lower than in the soluble protein group, which received 10 µg of purified NPt-VP1t. This unequal dosing, together with possible immunomodulatory effects of co-purifying bacterial proteins, may have influenced the immune responses observed in the IB group and should be considered when comparing the immunogenic potential of soluble versus insoluble forms [22]. Despite these limitations, comparison of the two formulations revealed clear immunogenic differences. The SP formulation induced significantly stronger humoral and cellular immune responses [23, 24] than the IB. Mice immunized with SP exhibited significantly higher VP1-specific IgG reactivity than those receiving the IB or PBS control (*p* < 0.05). After the first booster (week 4), antibody levels in the soluble group were approximately two-fold higher than in the insoluble group, peaking at week 8 and remaining elevated for at least two weeks post-booster. These findings are consistent with previous reports that effective antigen presentation by B lymphocytes and subsequent antibody production depend on adequate antigen dose and epitope integrity [22–24]. These results align with previous studies showing that solubility and proper folding enhance antigen uptake, processing, and presentation by antigen-presenting cells (APCs) [25, 26].

Cell-mediated immunity was assessed by splenocyte proliferation (Fig. 8). Upon VP1 stimulation, the SP group achieved a mean S.I. of 0.826 ± 0.104, which was approximately 1.6-fold higher than the IB group (0.53 ± 0.03) and significantly greater than both the IB and control groups (*p* < 0.05). This suggests more efficient antigen processing and presentation, likely due to better solubility, structural integrity, and epitope accessibility [26]. Soluble proteins are generally more efficiently internalized by APCs, resulting in stronger T-cell activation [27]. The poorer response in the insoluble group is likely due to aggregation and impaired epitope presentation [28]. The ConA-induced proliferation was similar across groups, indicating that baseline splenocyte reactivity was not altered by vaccination. These findings are consistent with reports that splenocyte proliferation correlates with effective cellular immune responses important for viral clearance [29].

Cytokine profiling confirmed that the soluble NPt-VP1t protein induced a balanced and robust Th1/Th2 response, with significantly elevated levels of IFN-γ and IL-2 (Th1 cytokines) and IL-4, IL-6, and IL-10 (Th2 cytokines) compared to insoluble protein and control groups (*p* < 0.05). The absence of increased TNF-α indicates that the immune response was not associated with excessive inflammation. IFN-γ is particularly important for antiviral defence and has been linked to protective immunity [30, 31]. The balanced Th1/Th2 cytokine profile observed here resembles responses induced by licensed subunit vaccines such as those for influenza and HPV [32]. These results agree with previous studies showing that VP1-based constructs can induce neutralizing antibodies and T-helper proliferation [33], although neutralizing reactivity vary with vaccine format [34]. An additional consideration is that all groups including the PBS control were immunized with formulations containing complete or incomplete Freund’s adjuvant. These adjuvants are known to induce potent inflammatory responses and may mask mild vaccine-related adverse effects. The lack of an adjuvant-free control limits our ability to conclude that the recombinant proteins were independently well-tolerated. Future studies should consider adjuvant-free formulations or alternative adjuvants suitable for human use. While the robust antibody and T-cell responses observed here indicate strong immunogenic potential, no viral challenge experiments or neutralizing antibody assays were performed. Future work should include viral neutralization assays and EV71 challenge studies to confirm protective efficacy. Overall, these findings reinforce the importance of protein solubility in subunit vaccine design and highlight the soluble NPt-VP1t protein as a promising candidate for further development against EV71.

## Conclusion

The soluble NPt-VP1t recombinant protein elicited strong humoral and cellular immune responses in adult BALB/c mice. Compared to the insoluble formulation, the soluble protein demonstrated significantly enhanced anti-VP1 IgG reactivity, splenocyte proliferation, and cytokine secretion. These results suggest that the soluble NPt-VP1t construct has promising immunogenic potential as a subunit vaccine candidate against EV71. However, further investigations including neutralization assays and viral challenge experiments are required to confirm its protective efficacy.

## Supplementary Information

Below is the link to the electronic supplementary material.

Supplementary Material 1
