# Supplementary material for: Soluble recombinant enterovirus 71 VP1 fused to truncated newcastle disease virus nucleoprotein elicits immune responses in mice
Source: Braz J Microbiol. 2025 Sep 23;56(4):2743–55. doi: 10.1007/s42770-025-01784-w (PMC12660611; doi:10.1007/s42770-025-01784-w)
Supplement: Supplementary file 1 — Supplementary Material 1 [file 42770_2025_1784_MOESM1_ESM.docx]

**Supplementary Fig S1**


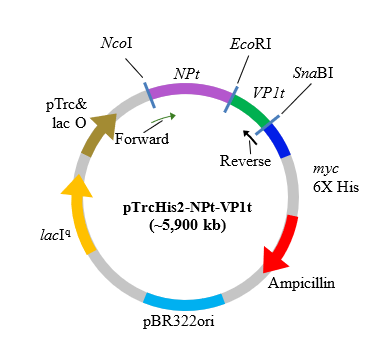


**Supplementary Figure S1. Schematic representation of the recombinant construct.**Plasmid map of pTrcHis2 encoding the truncated nucleocapsid protein (NPt; 1173 bp) of Newcastle disease virus (NDV) strain AF2240 fused to the truncated viral protein 1 fragment (VP1t; 300 bp, amino acids 198–297) of enterovirus 71 (EV71) strain MY104/9/SAR/97 [12]

**Supplementary Fig S2**


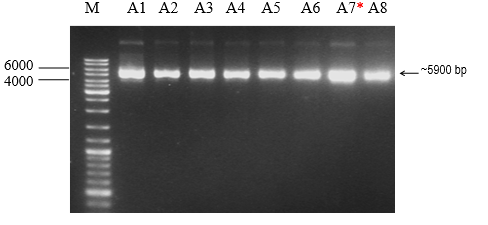


**Supplementary Figure S2. Verification of the extracted plasmid pTrcHis2-NPt- VP1ton 1% (w/v) TAE agarose gels.** Plasmid pTrcHis2-NPt-VP1t was extracted from *E. coli* Rosetta-gami using the HiYield Plasmid Mini Kit (Yeastern Biotech, Taiwan) following the manufacturer’s protocol. A single colony was cultured in 3 mL LB broth (1% casein peptone, 0.5% NaCl, 0.5% yeast extract) supplemented with 100 µg/mL ampicillin at 37 °C, 250 rpm overnight. Cells were harvested by centrifugation (14,000 × g, 1 min), and plasmids were isolated using PD1 buffer (with RNase A), PD2 lysis buffer, and PD3 neutralization buffer, followed by column purification and elution in 50 µL elution buffer. For verification, 5 µL of extracted plasmid DNA was mixed with 1 µL of 6× DNA loading dye Thermo Scientific (10mM Tris-HCL (pH 7.6) 0.03% bromophenol blue, 0.03% xylene cyanol FF, 60% glycerol and 60mM EDTA) and analyzed on 1% (w/v) agarose gels prepared in TAE buffer (40 mM Tris-acetate, 1 mM EDTA, pH 8.0). Electrophoresis was carried out at 80 V for 40 minutes, and gels were stained with 0.5 µg/mL ethidium bromide and visualized using the Gel Doc™ Imaging System (Bio-Rad, USA). M: DNA marker; A1–A8: plasmids from individual colonies. (*) indicates plasmid used for restriction digestion.

**Supplementary Fig S3**

**
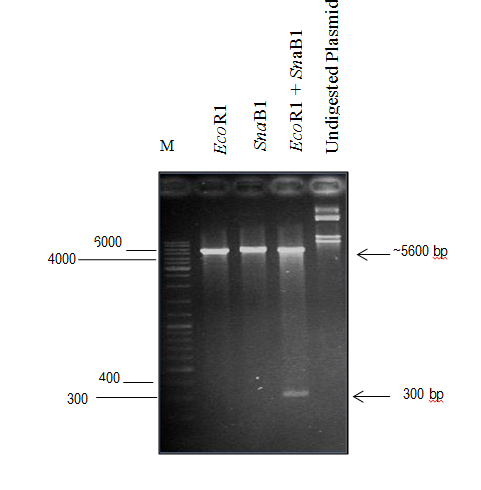
**

**Supplementary Figure S3. Restriction enzyme digestion of the pTrcHis2-NPt-VP1t plasmid and gel purified product using *Eco*R1 and *Sna*B1**. Plasmid DNA was digested with *Eco*RI and *Sna*BI (Thermo Scientific, USA) using FD buffer (Thermo Scientific, USA) according to the manufacturer’s instructions. Single and double digestions were performed in 10 µL reactions, and the products were resolved on 1% TAE agarose gels. Two distinct bands were observed following double digestion of the pTrcHis2-NPt-VP1t plasmid, confirming the presence of the insert. M: DNA marker.

**Supplementary Fig S4**


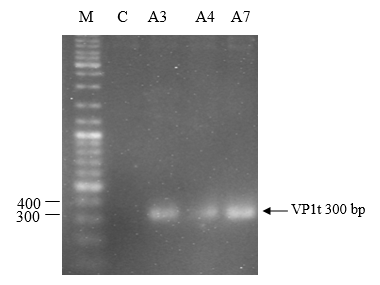


**Supplementary Figure S4. PCR screening of selected positive bacterial transformants.** PCR screening of positive bacterial transformants was performed using vector-specific primers (forward 5′-GCT ATA GAA TTC AGC GAG C GCT TAC CA-3′; reverse 5′-GC GAG TAC GTA AAG GGT AGT AAT GGC-3′) targeting the VP1t insert. Amplified products were resolved on 2% (w/v) TAE agarose gels. M: DNA marker; C: negative control (sterile water); A3, A4, A7: plasmids extracted from individual colonies.

**Supplementary Fig S5**


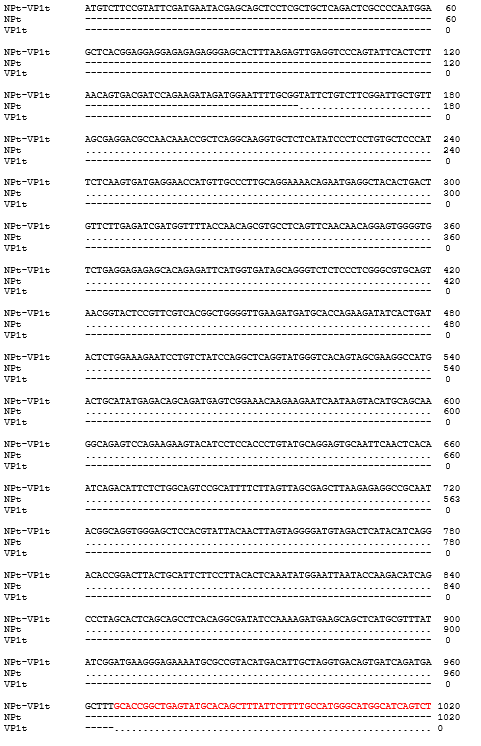

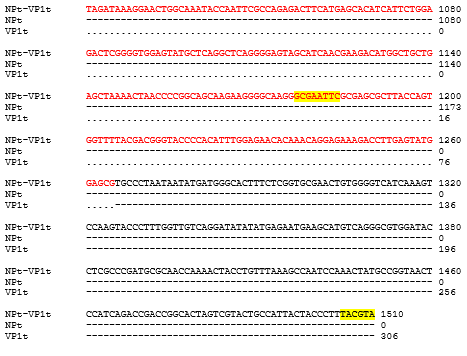


**Supplementary Figure S5. The PCR amplified sequences of *NPt-VP1t* cloned in *E. coli* Rosetta-gami aligned with the gene sequence of *NPt* and *VP1t* cloned in the *E. coli* TOP10 using BLAST.** Plasmid pTrcHis2-NPt-VP1t was sequenced by Sanger sequencing (Macrogen Inc., Korea), and the amplified sequences were aligned with reference NPt and VP1t sequences using BLAST, BioEdit, ClustalW2 (EMBL-EBI), and ExPASy tools. In the alignment, identical nucleotides are indicated by dots (·), mismatches by dashes (–), NPt sequences are shown in black, VP1t sequences in red, and restriction enzyme sites are highlighted in yellow.
